# A Department of Defense Laboratory Consortium Approach to Next Generation Sequencing and Bioinformatics Training for Infectious Disease Surveillance in Kenya

**DOI:** 10.3389/fgene.2020.577563

**Published:** 2020-09-25

**Authors:** Irina Maljkovic Berry, Wiriya Rutvisuttinunt, Logan J. Voegtly, Karla Prieto, Simon Pollett, Regina Z. Cer, Jeffrey R. Kugelman, Kimberly A. Bishop-Lilly, Lindsay Morton, John Waitumbi, Richard G. Jarman

**Affiliations:** ^1^Viral Diseases Branch, Walter Reed Army Institute of Research, Silver Spring, MD, United States; ^2^Office of Genomics and Advanced Technologies National Institute of Allergy and Infectious Diseases, Bethesda, MD, United States; ^3^Genomics & Bioinformatics Department, Biological Defense Research Directorate, Naval Medical Research Center-Frederick, Fort Detrick, MD, United States; ^4^Leidos, Reston, VA, United States; ^5^College of Public Health, University of Nebraska Medical Center, Omaha, NE, United States; ^6^Center for Genomic Studies, United States Army Medical Research Institute for Infectious Diseases, Frederick, MD, United States; ^7^Global Emerging Infections Surveillance, Armed Forces Health Surveillance Branch, Silver Spring, MD, United States; ^8^Basic Science Laboratory, US Army Medical Research Directorate-Africa/Kenya Medical Research Institute, Kisumu, Kenya

**Keywords:** NGS, bioinformatics, workshop, infectious disease, DoD

## Abstract

Epidemics of emerging and re-emerging infectious diseases are a danger to civilian and military populations worldwide. Health security and mitigation of infectious disease threats is a priority of the United States Government and the Department of Defense (DoD). Next generation sequencing (NGS) and Bioinformatics (BI) enhances traditional biosurveillance by providing additional data to understand transmission, identify resistance and virulence factors, make predictions, and update risk assessments. As more and more laboratories adopt NGS and BI technologies they encounter challenges in building local capacity. In addition to choosing the right sequencing platform and approach, considerations must also be made for the complexity of bioinformatics analyses, data storage, as well as personnel and computational requirements. To address these needs, a comprehensive training program was developed covering wet lab and bioinformatics approaches to NGS. The program is meant to be modular and adaptive to meet both common and individualized needs of medical research and public health laboratories across the DoD. The training program was first deployed internationally to the Basic Science Laboratory of the US Army Medical Research Directorate-Africa in Kisumu, Kenya, which is an overseas Lab of the Walter Reed Army Institute of Research (WRAIR). A week-long workshop with intensive focus on targeted sequencing and the bioinformatics of genome assembly (*n* = 24 participants) was held. Post-workshop self-assessment (completed by 21 participants) noted significant median gains in knowledge domains related to NGS targeted sequencing, bioinformatics for genome assembly, and sequence quality assessment. The participants also reported that the information on study design, sample preparation, sequencing quality control, data quality assessment, reporting, and basic and advanced bioinformatics analysis were the most useful information presented in the training. While longer-term evaluations are planned, the training resulted in significant short-term improvement of a laboratory’s self-reported wet lab and bioinformatics capabilities. This framework can be used for future DoD laboratory development in the area of NGS and BI for infectious disease surveillance, ultimately enhancing this global DoD capability.

## Introduction

Development of Next-Generation Sequencing (NGS), or High-Throughput Sequencing (HTS), has revolutionized life sciences, dramatically increasing the variety of questions that can be answered using genomic sequence data. With this continuously evolving and growing field, the need for adequate computational hardware resources, software, and expertise to analyze large and complex data is also increasing. The field of bioinformatics has thus experienced substantial growth and advancement in recent years, and the requirement for highly skilled and specialized personnel has surged.

Within the Department of Defense (DoD), NGS and bioinformatics are routinely used to answer many scientific and research questions that ultimately aid in protection of the armed forces, as well as the general population (Kijak et al., 2017; Colby et al., 2018; Ehrenberg et al., 2019; Waickman et al., 2019). Infectious diseases are one area where such research is of high importance. Like the general population, United States forces are vulnerable to many infections commonly occurring within the United States, such as influenza, coronavirus, adenovirus and antibiotic resistant bacterial infections including but not limited to infection by methicillin resistant *Staphylococcus aureus* (MRSA); pathogens that have the ability to negatively impact United States force readiness and mission goals (MacPherson et al., 1923; Beam et al., 1959; Earhart et al., 2001; Shanks and Hodge, 2011; Millar et al., 2017, 2019). In addition, global deployment of the United States forces also puts them at a higher risk for infections that occur more frequently outside the United States, such as Ebola, dengue, Zika, cholera, malaria, leishmaniasis, shigellosis, and many others (Riddle et al., 2011; Murray et al., 2015). The DoD Global Emerging Infections Surveillance (GEIS) program seeks to improve infectious disease surveillance, prevention, and response capability to better protect the health of the military force. Utilizing a global network of partner DoD medical research and public health laboratories, GEIS funds surveillance activities in over 70 countries to inform force health protection through timely and actionable infectious disease surveillance information (Chakhunashvili et al., 2017; Chang et al., 2018; Coleman et al., 2018; Koka et al., 2018; Anyamba et al., 2019; Guerra et al., 2019; Juma et al., 2019; Rivers et al., 2019; Rocha et al., 2019; Sugiharto et al., 2019). Unsurprisingly, development of NGS and bioinformatics methods for infectious disease surveillance and control has enabled a rapid expansion of GEIS partner studies that utilize pathogen genomic information (Frey et al., 2016; Maljkovic Berry et al., 2016, 2019a; Lee et al., 2017; Mullins et al., 2017; Salje et al., 2017; Cowell et al., 2018; LaBreck et al., 2018; Srijan et al., 2018; Grubaugh et al., 2019; Kim et al., 2019; Mbala-Kingebeni et al., 2019; Millar et al., 2019; Pollett et al., 2019; Wiley et al., 2019). However, NGS and bioinformatics can generally be technically challenging, as it requires specific knowledge of complex wet lab and bioinformatics processes (Maljkovic Berry et al., 2019b). Therefore, and in spite of great interest in this technology, only a few partner laboratories have been adequately equipped to utilize these approaches to their full potential.

**FIGURE 1:**
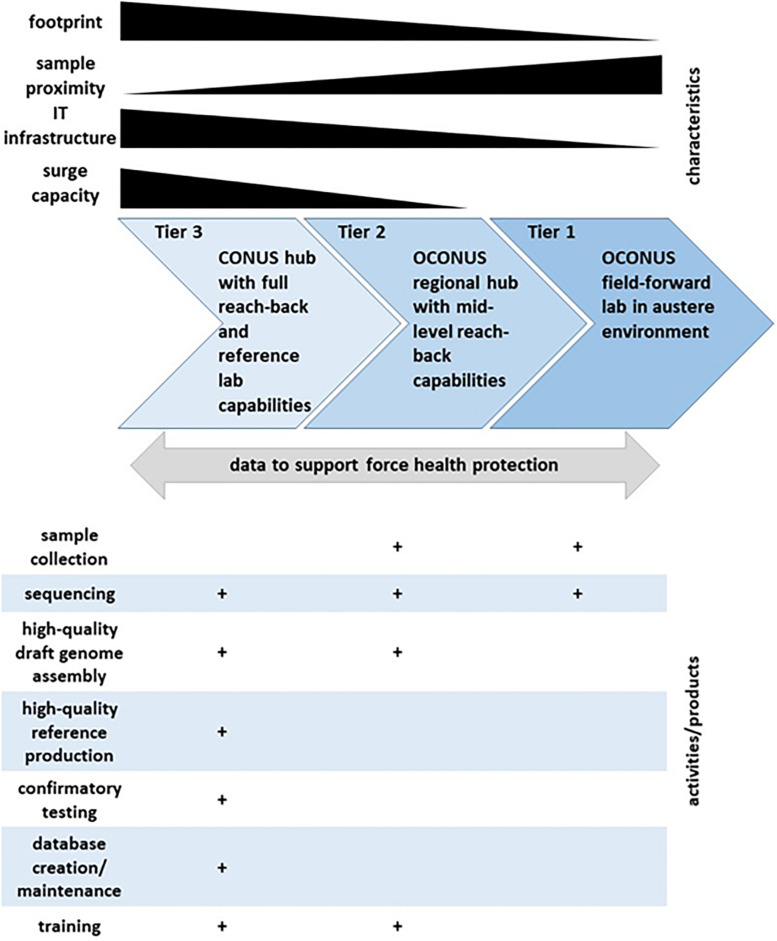
Tiered next generation sequencing (NGS) and bioinformatics (BI) capabilities for biosurveillance. Relative levels of laboratory and equipment footprint, proximity to source of biosurveillance samples, information technology (IT) infrastructure, and sequencing and bioinformatics surge capacity are displayed by black gradient bars along the **top**. Continuous flow of data back and forth among all three tiers is depicted by gray arrow, and expected types of activities and products by tier are illustrated by plus marks (+) along the **bottom**.

In 2017, GEIS created a Consortium to address the increasing needs and challenges associated with NGS and bioinformatics at DoD medical research and public health laboratories. The vision of the Consortium is to rapidly detect and characterize known, emerging, and novel infectious disease agents through establishment of a harmonized DoD laboratory NGS and bioinformatics capability to inform force health protection decision making. The Consortium today represents a network of DoD laboratories that use NGS and bioinformatics for infectious disease surveillance. A baseline assessment and initial training effort was led by GEIS and three DoD core sequencing and bioinformatics laboratories: WRAIR-VDB (Walter Reed Army Institute of Research-Viral Diseases Branch), NMRC-BDRD (Naval Medical Research Center-Biological Defense Research Directorate), and USAMRIID-CGS (United States Army Medical Research of Infectious Diseases-Center for Genome Science). The Consortium performed an assessment of the GEIS DoD laboratory partners with access to Illumina MiSeq or other NGS instrument(s), in order to evaluate existing laboratory capabilities in NGS and bioinformatics, and to map gaps and needs in laboratory utilization of these tools to meet their mission goals of infectious disease surveillance. Limited access to experienced and knowledgeable NGS and bioinformatics personnel was one of the main gaps, making basic and advanced bioinformatics analyses a common challenge across the network. Another challenge was the restrictive and limited informatics infrastructure, especially in some of the participating laboratories located in low-and-middle income countries (LMICs). However, the challenge of finding personnel with sufficient training in NGS and bioinformatics was not only observed in laboratories located in LMICs, it was also apparent in domestic laboratories, thus highlighting the need to develop a structured NGS and bioinformatics training for the specific needs of DoD biosurveillance programs. Such training would have to be standardized across the Consortium network, as well as made agile enough to meet different levels of needs and computational resources of the participating DoD laboratories. Using the baseline information from the assessment, desired sequencing capabilities for DoD research and public health laboratories were divided into three tiers (Figure 1). Here we present the deployment of NGS and bioinformatics training with our partner laboratory in Kenya, United States Army Medical Research Directorate – Africa (MRD-A). Future iterations of similar trainings and assessments will be used to further strengthen global infectious surveillance for DoD utilizing genomics and bioinformatics.

## Materials and Equipment

Samples used for the NGS hands-on training included dengue virus 2 (DENV-2) and chikungunya (CHIKV) and were provided on-site. Controls for library preparation, MiSeq sequencing and TapeStation for both DENV-2 and CHIKV were validated and prepared at VDB-WRAIR in the months prior to the planned NGS&BI training in Kenya. Prior to shipment of controls to Kenya, the control concentrations were measured and documented and the information was sent to MRD-A. Coordination of the reagent and control shipment from VDB-WRAIR to Kisumu, Kenya started a month prior to the training. Four Linux laptops and two Linux servers were prepared for hands-on bioinformatics training. A list of software was prepared by the Consortium and sent out to MRD-A Lab for installation onto the training computers. The software list included ngs_mapper, IGV, Geneious, MEGA7, EDGE (servers only) (Robinson et al., 2011; Kumar et al., 2016; Viral Diseases Branch WRAIR, 2016; Philipson et al., 2017). Three weeks prior to the training, a hands-on genome assembly training dataset was designed, consisting of dengue, chikungunya, and influenza raw fastq data, as well as hands-on performance instructions. The whole dataset was tested at VDB-WRAIR prior to training and saved onto the training computers.

## Methods

### Day 1

Lectures and theory included: History of sequencing, overview of NGS, library preparation, quantification, validation and pooling. In detail: (i) List of library preparation kits used by core DoD for different projects and specimens were highlighted; (ii) Several topics on types of kits for viruses, bacteria and parasite work were heavily discussed throughout the lecture; (iii) Specific library preparation kits were highlighted including TruSeq, QIASeq Fx, Kappa, NexteraXT, RNA Access and DNAFlex; (iv) AmpureXP Beads clean up after PCR reactions and library preparation was emphasized as preferred method; (v) Different library validations, including qPCR, Qubit and TapeStation were highlighted as essentials for quality control (QC); (vi) Library pooling based on TapeStation and Qubit were introduced; (vii) Two exercises of how to calculate amount of each library for pooling were conducted. Preparations were made for the upcoming bioinformatics training.

### Day 2

Hands-on training for NGS wet lab was performed with 24 participants. The participants were separated into two groups based on their NGS background and interests for hands-on performance. Group 1 prepared the NexteraXT library from the amplicons and assessed amplicons using both Qubit and TapeStation prior to NexteraXT library preparation. The NexteraXT libraries were validated using both Qubit and TapeStation. Group 2 validated the pooling based on the controls from the shipment and prepared sample sheets, the MiSeq instrument and PhiX controls. The libraries were loaded onto the Miseq. Bioinformatics training dataset was prepared on each computer. Server performance was tested for running the pipelines and tools needed for the training, and the training dataset analyses were executed to test functionality prior to the hands-on bioinformatics training.

### Day 3

Hands-on wet lab activities from Day 2 were summarized and any questions and concerns were addressed. Lectures on laboratory project experimental design (to include bioinformatics), bioinformatics data cleaning and pre-processing, and genome assembly through reference mapping were performed, as well as exercises in experimental design and genome consensus calling. For hands-on bioinformatics training, the 24 participants were divided into six different groups, each group utilizing one training computer or server. Ngs_mapper was used as the example of a reference mapping pipeline. The first training was performed on the DENV fastq dataset, including training on usage of different stages of the pipeline, setting a desired reference genome and running the pipeline. After ngs-mapper jobs were completed, interpretation of the output, how to utilize data quality scores and depth of coverage, how to assess the performance of the sequencing and the genome assembly were performed. Manual QC and genome curation were performed. The second training dataset consisted of CHIKV fastqs and was used for training on multiple reference usage and reference selection, in addition to repeating the above steps for dataset one.

### Day 4

Bioinformatics hands-on training was continued by evaluation of the CHIKV runs for reference genome selection. Based on the best reference choice, the reference mapping run was repeated. The repetition was incorporated on purpose to ensure better knowledge retention. Following reference mapping, the output of CHIKV assembly was evaluated and its genome curated. The data that were used for this training were purposefully chosen to be of lower quality, so that different challenges of genome assembly curation were highlighted, as well as the importance of QC and what consequences a lack of QC might result in. The last reference mapping analysis was performed on CHIKV data but now the participants learned how to change different pipeline thresholds, picking their own requirements for minimum base quality, consensus type output and the like. In addition, lectures were conducted covering theory of *de novo* genome assembly, assembly of bacterial genomes, and troubleshooting and maintenance of the MiSeq platform.

### Day 5

A summary of wet lab activities and library pooling to obtain optimal cluster density was presented. An exercise aimed at the evaluation of several MiSeq runs was performed. Management of sequencing libraries and data, and prevention of chimeric sequence data generation and mislabeling were discussed. Bioinformatics training on the influenza dataset was performed separately since influenza virus has a segmented genome and bioinformatically, full genome assembly is slightly more complicated. How to recognize presence of influenza reassortment was covered. A workshop survey was distributed (Supplementary Material) and the workshop was concluded.

## Results

### NGS and Bioinformatics Training Modules

**FIGURE 2:**
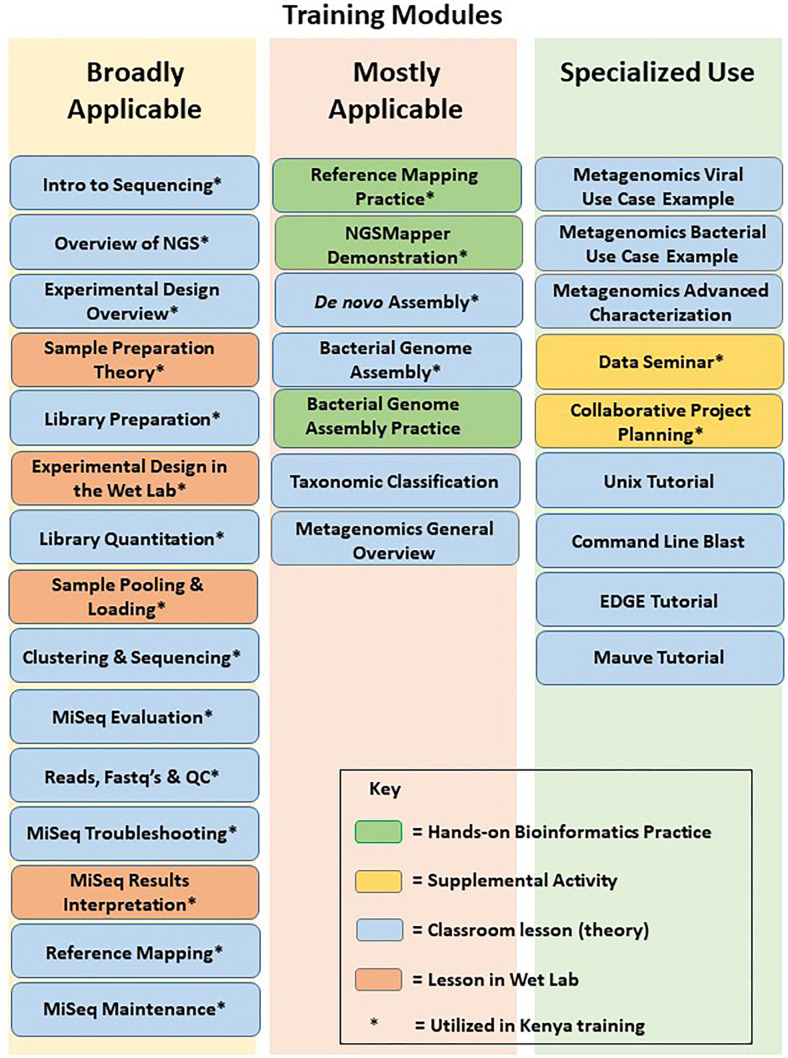
NGS and bioinformatics training modules. Modules used in training of MRD-A are denoted with an asterisk.

A comprehensive training curriculum was constructed that consisted of standardized wet lab and bioinformatics theory modules (Figure 2) as well as hands-on training. The modules could be independently compiled into a set of theoretical lectures that could be adjusted for the existing laboratory tiers and specific knowledge gaps. As they were designed to meet the particular DoD surveillance needs, the modules were divided into two main wet lab sequencing and two main bioinformatics analyses approaches. The wet lab lectures could thus be adjusted to cover: (i) the theory of targeted sequencing, which is mainly used in response to epidemics and outbreaks of known pathogens; and (ii) the theory of metagenomics, which is usually used for pathogen discovery and identification. The bioinformatics lectures focused on: (i) the genome assembly and curation analyses, an essential part of outbreak genomic surveillance; and (ii) the bioinformatics of pathogen discovery, usually the most challenging aspect of basic sequencing-based biosurveillance. In addition to these, modules covering other parts of NGS and bioinformatics were included, such as theory of experimental design, troubleshooting, and equipment maintenance. The theory modules were complemented with development of corresponding hands-on wet lab and bioinformatics training of the above approaches.

### NGS and Bioinformatics Training Deployment

**FIGURE 3:**
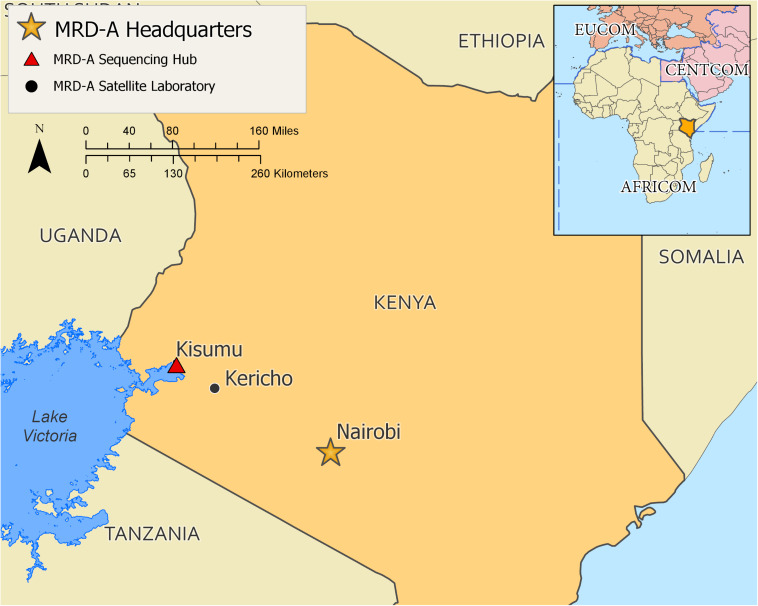
A map of training performance site and participating partner laboratories from Kenya. Red triangle shows where the training was held.

Based on the results of the initial laboratory assessment, training was recommended for the GEIS partner US Army Medical Research Directorate – Africa (MRD-A) laboratories in Kenya. For MRD-A’s initial needs, which mainly cover sequencing and analyses of known pathogen outbreaks and epidemics in the region, a 1 week on-site workshop was constructed where the wet lab targeted sequencing was covered in both lectures (specific assembled modules) and hands-on practice, followed by bioinformatics theory (specific assembled modules) and hands-on practice of pathogen genome assembly and curation (Figure 2). This approach was specifically designed based on the needs and gaps that were highlighted during the initial assessment of MRD-A capabilities. Participating in the training were representatives from various MRD-A and Kenya Medical Research Institute (KEMRI) laboratory divisions in Kenya: Basic Science, Viral Hemorrhagic Fevers, Entomology, Flu Lab, Antimicrobial Resistance, Sexually Transmitted Infections, Microbiology Hub-Kericho, Influenza, and KEMRI-Centers for Disease Control divisions (Figure 3). There was a total of 24 workshop participants.

We undertook a rapid evaluation of participants’ self-reported baseline and post-workshop knowledge across ten skill domains related to genomic sequencing (Supplementary Material). We also determined individual-level gains in self-reported knowledge after completing the workshop. This was measured with a single hard-copy questionnaire administered after the workshop. This survey asked the participants to self-rate their knowledge in each skill domain on a customized scale of 1–10 (1 = “no prior knowledge”, 10 = “high level of experience”) before and after the workshop. Median baseline and post-workshop scores are presented in Table 1. While interpretation of these metrics is limited due to the subjectivity of the self-reported knowledge measurements, particularly when measured at a single point in time, the IQR and range around the median reported knowledge scores did suggest that this sample of participants had varying expertise across each of these skill domains. Pre-training baseline scores suggested that the participants had, in particular, less self-reported expertise in NGS library validation, Illumina MiSeq run validation, experimental design for bioinformatics analysis, and FASTQ data cleaning and pre-processing.

**TABLE 1:** Self-reported knowledge across skill domains of genomic sequencing (*n* = 21 respondents).

Knowledge domain	Pre-workshop score^a^	Post-workshop score^a^	Post-workshop gains	
	Median (IQR)	Median (IQR)	Median (IQR)	*p*-value^b^
NGS technology	4 (3,5)	7 (6,8)	3 (2,3)	<0.001
Illumina MiSeq sequencing chemistry	4 (2,5)	7 (6,8)	3 (2,4)	<0.001
NGS library preparation	5 (2,6)	8 (7,9)	3 (2,4)	<0.001
NGS library validation	2 (1,5)	8 (6,8)	4 (3,4)	<0.001
MiSeq run validation	2 (1,3)	6 (4,8)	3 (2,4)	<0.001
Experimental design for bioinformatics analysis	2 (1,4)	6 (5,8)	3 (2,4)	<0.001
FASTQ data cleaning and pre-processing	2 (1,5)	6 (5,8)	4 (2,4)	<0.001
Reference mapping	3 (1,5)	7 (6,9)	4 (1,5)	<0.001
Linux OS use and command line	2 (1,5)	5 (3,7)	1 (0,3)	<0.001
Consensus sequence calling and manual curation	2 (1,3)	6 (5,8)	4 (2,5)	<0.001

*NGS, next generation sequencing; OS, operating system. ^a^Maximum possible score is 10. ^b^Derived from Wilcoxon signed rank test.*

There were substantial gains in self-reported knowledge across all skill domains (Table 1), with the notable exception of Linux OS and command line skills, suggesting that this is a particular area of residual training need. Indeed, Linux OS and command line skill had the lowest post-workshop self-reported knowledge scores. A module was later developed specifically to fill this gap (Figure 2). The questionnaire also measured the participants’ perceptions on the most “useful” information learned during the NGS library and bioinformatics components of the workshop. This was measured by free-text open ended questions (Table 2).

**TABLE 2:** Information reported by participants to be the most useful (*n* = 21 respondents)^a^.

	*n*	%^b^
NGS library preparation	12	60
NGS library validation	7	35
Sample pooling	6	30
Tapestation use	5	25
Library normalization	5	25
Qubit use	2	10
QC for sequence reads	1	5
Experimental design considerations	1	5
Sequencing platform overview	1	5
MiSeq runs (hands-on experience)	1	5
Sample pre-processing	1	5
Nextera-XT protocol	1	5
MiSeq run troubleshooting	1	5
Genome assembly (reference mapping and curation)	15	71
Sequence read QC	8	38
*De novo* sequencing	4	19
Experimental considerations	3	14
Software (including NGS_mapper and IGV)	3	14
Mapping bacterial sequences	1	5
Output analysis	1	5

*^a^Derived from open questions: “What information was most useful to you that this NGS library provided?” and “What information was most useful to you that this bioinformatic workshop provided?”. ^b^Some participants indicated > 1 item of information in response. QC, quality control.*

The participants were also asked in which topics they felt they would like more training and experience (Table 3) and how to improve future iterations of this workshop (Table 4). The participant’s responses all highlight the complexity and the diversity of considerations within NGS and bioinformatics. The many topics that can be covered and trained upon for the fields of infectious disease surveillance and control alone, and the associated time that it would take to train and educate the workforce, would indicate a large gap in the currently existing education programs.

**TABLE 3:** Suggested topics for more training/experience, as reported by participants (*n* = 21 respondents)^a^.

	*n*	%^b^
Phylogenetics and other advanced bioinformatics analysis	8	38
Metagenomics for pathogen discovery	4	19
De novo assembly	4	19
Linux OS/cluster	4	19
MiSeq loading and run evaluation	3	14
Bacterial genomics	3	14
Pipeline development (including open source bioinformatic tools)	3	14
Sequence assembly	2	10
Read QC	2	10
Library prep	1	5
16s and 18s molecular analysis	1	5
Sample pre-processing	1	5
Bioinformatic experimental design	1	5
SNP detection and variant calling	1	5
Sample sheet prep	1	5
Reference mapping	1	5
Plasmid sequencing	1	5
Recombination detection	1	5
Comparative genomics	1	5
Outbreak investigations	1	5

*^a^Derived from open question: “What topic would you like more training/experience in (if any)?”. ^b^Some participants indicated more than one line item of information. QC, quality control.*

**TABLE 4:** Participants’ suggestions for workshop improvements (*n* = 21 respondents)^a^.

	*n*	%^b^
More time (longer workshop >1 week)	4	19
More hands on training/practical sessions (less theory/slides)	4	19
More time on bioinformatic data interpretation and analysis	3	14
Increased frequency of workshop with follow-up training	2	10
More wet lab time	2	10
More laptops	1	5
Split into beginner and advanced classes	1	5

*^a^Derived from open question: “Suggestions for workshop improvement?”. ^b^Some participants had >1 suggestion.*

## Discussion

The rapid growth and utility of NGS and bioinformatics for research and biosurveillance has resulted in the emergence of DoD requirements for implementation of sequencing and computational technologies, as well as access to highly trained and knowledgeable personnel in the fields of NGS and bioinformatics. Specifically the latter point remains one of the major challenges across the DoD, and even though bioinformatics programs have more recently gained larger momentum in academia, lack of workforce with early-on and/or specialized bioinformatics training is still palpable in the government settings, particularly in government labs outside the continental United States. Therefore, NGS and bioinformatics training programs for infectious disease surveillance have recently been developed by many government agencies or non-governmental organizations. Within the United States Government, Canada, and the European Union, there is movement towards training and coordinated promotion of standardized quality assurance and quality control practices for pathogen genome sequencing using NGS technologies (e.g., Illumina) (Cui et al., 2015; Gargis et al., 2016; Nadon et al., 2017). Some recent examples include the GenomeTrakr program at the Food and Drug Administration, Next Generation PulseNet at Centers for Disease Control, and the Global Microbial Identifier for food-borne pathogen surveillance (Moran-Gilad et al., 2015; Timme et al., 2018; Ribot et al., 2019). More recently, the SARS-CoV-2 Sequencing for Public Health Emergency Response, Epidemiology, and Surveillance (SPHERES) national genomics consortium was set up by the Centers for Disease Control, to coordinate SARS-CoV-2 sequencing across the United States (Centers for Disease Control and Prevention, 2019). Within the DoD, the training designed and implemented by the GEIS Consortium aims to develop lasting and sustainable capabilities for pathogen genomic sequencing and bioinformatics at DoD medical research and public health laboratories in overseas locations.

Our experience in deploying a comprehensive yet customizable classroom and hands-on training in NGS and bioinformatics in Kenya was overall successful (see caveats of assessment below) and is a potential model for future training programs in similar environments. This training program consisted of foundational material in sequencing theory and experimental design which formed a basis for more applied modules in targeted sequencing and metagenomics. Additionally, hands-on NGS wet lab and bioinformatics modules were further tailored to meet the needs of the laboratory participants using information obtained from a baseline landscape assessment. This training shows that a highly modular and deployable set of NGS and bioinformatics workshop components can be used within the DoD network of medical research and public health laboratories to improve sequencing wet lab capability, and analysis and interpretation of pathogen genomic data gathered using NGS and bioinformatics.

Embedded within this training workshop was a post-self-assessment questionnaire to gauge immediate improvements in knowledge gained from the workshop materials. It is important to note that this questionnaire has several limitations including a small sample size, the immediate nature of the assessment tool which does not allow one to measure long-term benefits, and the fact that the assessment was only delivered through written evaluation and self-report. Further, more objective measurements of knowledge and skill gains after workshops may not directly translate into effective implementation and retention of these skills. The latter requires medium and longer term evaluations in an implementation science framework (Nilsen, 2015). However, these data do suggest that the participants have perceived that this workshop offered productive training which has led to substantial gains in knowledge. In similar bioinformatics trainings in LMICs, technological limitations were identified as an impediment to knowledge acquisition and long-term improvements in bioinformatics capability (Pollett et al., 2016). This training attempted to overcome these barriers by (a) providing training laptops, (b) providing recommendations for IT upgrades, bioinformatics software, and computer networking, and (c) upgrading local IT equipment for bioinformatics during the workshop.

Following this workshop a mechanism to facilitate reach back support with embedded long-term training and mentorship has been instituted to overcome challenges associated with long-term sustainability of a sequencing capability at MRD-A. Included in this 5-year NGS and bioinformatics implementation plan for MRD-A are: (i) continuous contact and support by the core DoD sequencing laboratories, (ii) repetition of training with focus on real data and troubleshooting, (iii) additional hands-on training in other wet lab and bioinformatics approaches to achieve capability diversification, (iv) development of local computational infrastructure for bioinformatics, and (v) regular assessments of wet lab and bioinformatics knowledge retention. Laboratory-level assessments of proficiency and skill retention 1–2 years post-training have included external review of raw sequence data and consensus genomes generated from GEIS funded surveillance projects. We also anticipate deploying periodic blinded panel of samples or data files for follow-up assessments of knowledge retention and capability development. At the end of this period, the goal is to achieve a high quality diversified portfolio of NGS and bioinformatics capabilities at the site, which then may serve as a central DoD hub for sequencing and advanced characterization of Force Health Protection (FHP) relevant pathogens in Africa.

The current COVID-19 pandemic has further highlighted the importance of access to the NGS and bioinformatics in laboratories throughout the world. This makes the need of workshops such as ours even greater. However, the pandemic has also made travel and in-person learning a challenge, and therefore, GEIS is planning on development of virtual versions of the workshops to continue development of this important DoD-wide capability. In addition, Oxford Nanopore’s MinION platform has increasingly been used in pathogen outbreak studies for real-time in-field analyses throughout the world, including analyses of SARS-CoV-2 (Quick et al., 2016; Faria et al., 2018; Moore et al., 2020). Although training in the wet-lab and bioinformatics of this approach was not included in the workshop in Kenya to maintain simplicity and focus, the plan is to apply the modular approach for development and incorporation of a general DoD MinION-focused training for the GEIS partner laboratories. Currently, GEIS has established a separate MinION working group, and has been working in providing basic training in this technology to a subset of partner laboratories.

More broadly, the Consortium goal is the establishment of basic proficiencies and adopted norms in quality assurance and quality control in targeted (hybridization- or amplicon-based) and metagenomic sequencing for viral and bacterial pathogens leading to more reliable results which will ultimately improve DoD public health surveillance and response. An additional objective is the development and maintenance of advanced genomics and bioinformatics capabilities in the United States and priority overseas locations, in order to enhance global health surveillance and facilitate faster response to infectious disease outbreaks. Development of these capabilities with GEIS DoD laboratory partners will require sustained commitment and global coordination. The end results will be the ability to reliably and rapidly sequence, identify, and characterize pathogens of public health importance in order to improve biosurveillance efforts and inform FHP measures throughout the world.

## Data Availability Statement

All datasets presented in this study are included in the article/Supplementary Material.

## Author Contributions

IM, WR, KB-L, LM, and RJ designed the training modules and the workshop modules. IM, WR, LV, LM, KP, RJ, JW, SP, and RC prepared and instructed the workshop. IM, WR, LV, KP, SP, RC, JK, KB-L, LM, JW, and RJ performed workshop post-assessment and wrote the manuscript. All authors contributed to the article and approved the submitted version.

## Disclaimer

The views expressed in this article are those of the authors and do not necessarily reflect the official policy or position of the Department of the Army, Department of the Navy, Department of Defense, or United States Government. Several of the authors are United States Government employees. This work was prepared as part of their official duties. Title 17 U.S.C. §105 provides that “Copyright protection under this title is not available for any work of the United States Government.” Title 17 U.S.C. §101 defines a U.S. Government work as a work prepared by a military service member or employee of the U.S. Government as part of that person’s official duties.

## Conflict of Interest

LV and RC were employed by company Leidos, United States. The remaining authors declare that the research was conducted in the absence of any commercial or financial relationships that could be construed as a potential conflict of interest.
