# Supplementary material for: A Department of Defense Laboratory Consortium Approach to Next Generation Sequencing and Bioinformatics Training for Infectious Disease Surveillance in Kenya
Source: Front Genet. 2020 Sep 25;11:577563. doi: 10.3389/fgene.2020.577563 (PMC7546821; doi:10.3389/fgene.2020.577563)
Supplement: Supplementary file 1 [file Data_Sheet_1.PDF]

Office Use Only:

The USAMRU-K Sequencing and Bioinformatics and Workshop, 2018

The Next Generation and Sequencing Bionformatics Consortium (NGSBIC) is pleased to offer a Sequencing and Bioinformatics Workshop to be held in November 2018 at the US Army Research Unit-Kenya. To assist us in best tailoring this workshop to your needs, please complete this anonymous pre-workshop questionnaire

1. Do you need to sequence pathogen nucleotide data as part of your current research or public health activities?

- ☐ Yes
- ☐ No

2. Have you performed any pathogen sequencing before (targeted or unbiased)?

- ☐ Yes
- ☐ No

3. Please indicate which molecular diagnostic, sequencing methods/platforms you have used previously

- ☐ Conventional PCR
- ☐ Real-time PCR (diagnostic including qualitative or quantitative)
- ☐ Sanger sequencing
- ☐ Next Generation sequencing – e.g. Illumina (MiSeq, HiSeq, Novaseq), Life Technologies (Ion Torrent, SOLiD)

4. Which pathogens do you plan to sequence with currently or in the near future?

- ☐ RNA viruses, e.g. flaviviruses, influenza, HIV (specifiy)

  ____
  ____
  ____
  ____
  ____
- ☐ DNA viruses, e.g. CMV, EBV, adenovirus (specify)

  ____
  ____
  ____
- ☐ Bacteria
- ☐ Fungi
- ☐ Rickettsia
- ☐ Parasites
- ☐ Metagenomics/pathogen discovery
- ☐ Other, specify -

  ____
  ____

5. Please indicate, to the best of your knowledge, which sequencing capacity you currently have access to

- ☐ Sanger sequencing
- ☐ Next Generation sequencing wet-lab platforms - Illumina (MiSeq, HiSeq)
- ☐ Next Generation sequencing wet-lab platforms - Life Technologies (Ion Torrent)
- ☐ Post wet-lab bioinformatics pipeline for sequence quality check and curation
- ☐ Linux cluster
- ☐ Consultant bioinformatician
- ☐ Consultant software developer/programmer

6. Which of the following NGS bioinformatics software have you used (prior workshop)?

- ☐ Geneious or other sequence alignment software/pipelines, specify ______________________________
- ☐ IGV or other NGS output visualization software, specify ______________________________
- ☐ NGS output sequence assembly software/pipelines, specify ______________________________
- ☐ NGS pathogen discovery software/pipelines, specify ______________________________
- ☐ Other, specify ______________________________

7. Which of the following bioinformatics software do you have installed (prior workshop)?

- ☐ Geneious or other sequence alignment software/pipelines, specify ______________________________
- ☐ IGV or other NGS output visualization software, specify ______________________________
- ☐ NGS output sequence assembly software/pipelines, specify ______________________________
- ☐ NGS pathogen discovery software/pipelines, specify ______________________________
- ☐ Other, specify ______________________________

8. Which of the following operating systems do you have experience in (prior workshop)?

- ☐ Windows
- ☐ Unix/Mac
- ☐ Linux

9. Please indicate which of the following programming languages, if any, you are comfortable with

- ☐ Bash
- ☐ Python
- ☐ Perl
- ☐ R
- ☐ None
- ☐ Other, specify ______________________________

12. Please describe what you hoped to get out of this sequencing and bioinformatics workshop:

- ☐ Next-Generation sequencing wet-lab expertise (running specimens)
- ☐ Dry-lab pipeline expertise (interpreting NGS output for quality and genome assembly)
- ☐ Metagenomics expertise, inc. pathogen discovery
- ☐ Other, specify

____
____
____
____

Please answer following questions on a scale 1 to 10, with 1 being no prior knowledge, and 10 being high level of experience:

a) How much knowledge would you say you have in Next-generation sequencing (NGS) technology?
Prior workshop: 1 2 3 4 5 6 7 8 9 10
After workshop: 1 2 3 4 5 6 7 8 9 10

b) How much knowledge would you say you have in Illumina MiSeq sequencing chemistry?
Prior workshop: 1 2 3 4 5 6 7 8 9 10
After workshop: 1 2 3 4 5 6 7 8 9 10

c) How much knowledge would you say you have in NGS library preparation?
Prior workshop: 1 2 3 4 5 6 7 8 9 10
After workshop: 1 2 3 4 5 6 7 8 9 10

d) How much knowledge would you say you have in NGS library validation?
Prior workshop: 1 2 3 4 5 6 7 8 9 10
After workshop: 1 2 3 4 5 6 7 8 9 10

e) How much knowledge would you say you have in MiSeq run evaluation?
Prior workshop: 1 2 3 4 5 6 7 8 9 10
After workshop: 1 2 3 4 5 6 7 8 9 10

f) How much knowledge would you say you have in experimental design for bioinformatics analyses?
Prior workshop: 1 2 3 4 5 6 7 8 9 10
After workshop: 1 2 3 4 5 6 7 8 9 10

g) How much knowledge would you say you have in fastq data cleaning and preprocessing?
Prior workshop: 1 2 3 4 5 6 7 8 9 10
After workshop: 1 2 3 4 5 6 7 8 9 10

h) How much knowledge would you say you have in reference mapping?
Prior workshop: 1 2 3 4 5 6 7 8 9 10
After workshop: 1 2 3 4 5 6 7 8 9 10

i) How much knowledge would you say you have in Linux operating system usage and command line?
Prior workshop: 1 2 3 4 5 6 7 8 9 10
After workshop: 1 2 3 4 5 6 7 8 9 10

j) How much knowledge would you say you have in consensus sequence calling and manual curation?

Prior workshop: 1 2 3 4 5 6 7 8 9 10
After workshop: 1 2 3 4 5 6 7 8 9 10

12. What information was most useful to you that this NGS library workshop provided?

13. What information was most useful to you that this bioinformatics workshop provided?

14. What topic would you like more training/experience in (if any)?

15. Suggestions for workshop improvement and other comments
